# Fusion of X-Ray Images and Clinical Data for a Multimodal Deep Learning Prediction Model of Osteoporosis: Algorithm Development and Validation Study

**DOI:** 10.2196/70738

**Published:** 2025-09-18

**Authors:** Jun Tang, Xiang Yin, Jiangyuan Lai, Keyu Luo, Dongdong Wu

**Affiliations:** 1 Department of Information, Daping Hospital, Army Medical University, No.10 Daping Changjiang Branch Road, Yuzhong District, Chongqing, China; 2 Department of Orthopedics, Daping Hospital, Army Medical University, Chongqing, China; 3 Department of Traumatic Surgery, School of Basic Medicine, Army Medical University, Chongqing, China

**Keywords:** osteoporosis, chest X-ray, convolutional neural network, multimodal, attention mechanism, wavelet transform, soft attention

## Abstract

**Background:**

Osteoporosis is a bone disease characterized by reduced bone mineral density and mass, which increase the risk of fragility fractures in patients. Artificial intelligence can mine imaging features specific to different bone densities, shapes, and structures and fuse other multimodal features for synergistic diagnosis to improve prediction accuracy.

**Objective:**

This study aims to develop a multimodal model that fuses chest X-rays and clinical parameters for opportunistic screening of osteoporosis and to compare and analyze the experimental results with existing methods.

**Methods:**

We used multimodal data, including chest X-ray images and clinical data, from a total of 1780 patients at Chongqing Daping Hospital from January 2019 to August 2024. We adopted a probability fusion strategy to construct a multimodal model. In our model, we used a convolutional neural network as the backbone network for image processing and fine-tuned it using a transfer learning technique to suit the specific task of this study. In addition, we introduced a gradient-based wavelet feature extraction method. We combined it with an attention mechanism to assist in feature fusion, which enhanced the model’s focus on key regions of the image and further improved its ability to extract image features.

**Results:**

The multimodal model proposed in this paper outperforms the traditional methods in the 4 evaluation metrics of area under the curve value, accuracy, sensitivity, and specificity. Compared with using only the X-ray image model, the multimodal model improved the area under the curve value significantly from 0.951 to 0.975 (*P*=.004), the accuracy from 89.32% to 92.36% (*P*=.045), the sensitivity from 89.82% to 91.23% (*P*=.03), and the specificity from 88.64% to 93.92% (*P*=.008).

**Conclusions:**

While the multimodal model that fuses chest X-ray images and clinical data demonstrated superior performance compared to unimodal models and traditional methods, this study has several limitations. The dataset size may not be sufficient to capture the full diversity of the population. The retrospective nature of the study may introduce selection bias, and the lack of external validation limits the generalizability of the findings. Future studies should address these limitations by incorporating larger, more diverse datasets and conducting rigorous external validation to further establish the model’s clinical use.

## Introduction

### Background

Osteoporosis is a common metabolic bone disease characterized by decreased bone mineral density (BMD) and thinning of the trabecular structure of bone, leading to fragile bones and an increased risk of fracture. As the global population ages, osteoporosis has become a key factor affecting the health of middle-aged and older adults. However, the early symptoms of osteoporosis are not obvious, and most patients are diagnosed only after a fracture occurs, leading to increased complications and mortality [1]. Therefore, how to effectively identify people at high risk of osteoporosis and take timely preventive measures has become the focus of current clinical research.

Automated detection and classification of osteoporosis using deep learning (DL) has become a popular research topic in recent years [2]. Traditional prediction methods for osteoporosis mainly rely on a single data type, such as assessing BMD and classifying the degree of bone loss based on clinical data [3-5], computed tomography [6,7], magnetic resonance imaging [8], and X-ray images [9-12]. Dual-energy X-ray absorptiometry (DXA) is an internationally recognized method of measuring BMD and is also considered the gold standard for the diagnosis of osteoporosis [13]. However, DXA is less available and the cost of screening is high. For this reason, opportunistic screening offers a viable solution, as it uses clinical data for other indications without additional costs, radiation exposure, or patient time. In contrast, DL algorithm–based assessment of osteoporosis from X-ray images is a low-cost alternative to DXA [14-16]. Chest X-ray imaging has accumulated a large amount of imaging resources in routine applications such as pneumonia screening and bronchial disease examination, providing a natural imaging basis for opportunistic screening of osteoporosis. In addition, chest X-rays [11,17,18] can capture key bone structures affected by osteoporosis, such as ribs, thoracic vertebrae, and clavicles, further highlighting their application value in osteoporosis screening. Although such DL models based on a single data type have improved the accuracy and efficiency of osteoporosis diagnosis to a certain extent, the single data type only provides 1D information, neglecting the comprehensive consideration of information about the bone microenvironment, physiological characteristics, and patients’ clinical data, which leads to the prediction results that may not be comprehensive and accurate.

However, in the actual diagnostic process, doctors will comprehensively refer to various information sources, including medical image data, laboratory examination data, and medical record reports, to improve the accuracy of diagnosis [19]. This concept is introduced into DL, which aims to simulate the complex process of medical differential diagnosis through multimodal [20,21] data fusion. The fusion of multimodal medical data has become a hot research topic in interdisciplinary fields. Multimodal fusion can provide a more accurate description of samples, and it exploits the complementary and synergistic nature of each modality’s information more effectively than any single modality data [22]. As a result, multimodal fusion techniques have been widely used in several medical fields, including oncology, cardiovascular medicine, and radiology [23-25]. In the field of orthopedics, relevant multimodal studies are rapidly emerging [26,27], providing new ideas for osteoporosis prediction.

### Objectives

In this study, we propose a multimodal prediction model that fuses chest X-ray images and clinical data for opportunistic screening of osteoporosis. In the model construction, we used a convolutional neural network (CNN) as the backbone network for image processing and fine-tuned it to the specific task of this study using transfer learning techniques. In addition, we introduced a gradient-based wavelet feature extraction method. We combined it with an attention mechanism to assist in feature fusion, which strengthened the model’s focus on key regions of the image and further enhanced the model’s ability to extract image features.

## Methods

### Ethical Considerations

The study was approved by the institutional review board at the Chongqing Daping Hospital (2024_335). A waiver of informed consent was provided, as this retrospective study used existing information. Privacy and confidentiality of all patient data were maintained throughout the entire study. No compensation was provided to patients, as this study only involved the review of retrospective data.

### Dataset and Study Population

This study retrospectively collected multimodal data, including chest X-ray images and clinical data, from January 2019 to August 2024. The specific inclusion criteria were as follows: (1) patient age ≥50 years and (2) chest X-ray and DXA were performed on the same day. The specific exclusion criteria were as follows: (1) image data with artifacts, blurriness, or inability to display bone structures; (2) patients with other serious diseases that may affect bone health or interfere with diagnosis, such as malignant tumors and severe metabolic diseases; (3) patients who have recently used drugs that may affect bone metabolism or BMD measurement, such as hormone drugs and antiosteoporosis drugs; and (4) cases where the missing rate of clinical data reached or exceeded 30%. On the basis of these criteria, a total of 1780 cases met all the criteria, with 990 (55.62%) cases in the osteoporosis group and 790 (44.38%) cases in the nonosteoporosis group. Therefore, we extracted chest X-ray images (DICOM format) from the hospital’s communication system repository and collected the clinical data of patients from the back-end database, including age, sex, height, weight, white blood cell count, platelet count, serum calcium level, fasting blood glucose, and chest X-ray descriptive information. The model was trained through a 5-fold cross-validation.

The diagnosis of osteoporosis was based on DXA BMD measurement results. DXA negative means BMD is within the normal range, corresponding to the nonosteoporosis group. DXA positivity refers to a BMD lower than the normal range, corresponding to the osteoporosis group. The DXA manufacturer of Chongqing Daping Hospital is GE Lunar Prodigy model (Madison, WI, USA).

### Preprocessing

The DICOM format X-ray images were converted to the 8-bit PNG format to adapt to the DL frameworks. After conversion, the images were all manually reviewed. The image intensity was reversed to ensure no negative values and then normalized. All images were squared using zero padding and then downsampled to 224×224 pixels to fit a pretrained CNN on ImageNet. Using dynamic data augmentation to limit overfitting, this included random flipping (horizontal and vertical), random rotations (90° rotation), random translations (10% variation in each direction), random zooming (10% variation), and random contrast adjustment (factor of 0.3).

For the handling of missing values in clinical data, regarding white blood cell count and serum calcium level, since the numerical distribution of these features was roughly normally distributed, the mean of the variables was used for filling, which could better reflect the overall level. For features such as BMI, platelet count, and fasting blood glucose, as their numerical distributions might be skewed, the median of the variables was chosen for imputation, which was more robust in this case.

This research dataset included 990 positive (osteoporosis) and 790 negative (nonosteoporosis) samples, with a positive-to-negative ratio of 55.6% and 44.4%, respectively. In response to the mild imbalance characteristics of the dataset, this study adopted class weighting techniques to weight the loss function based on the distribution of training dataset classes, thereby improving the model’s sensitivity to identifying minority classes.

### Proposed Multimodal Model

#### Overview

**Figure 1.**
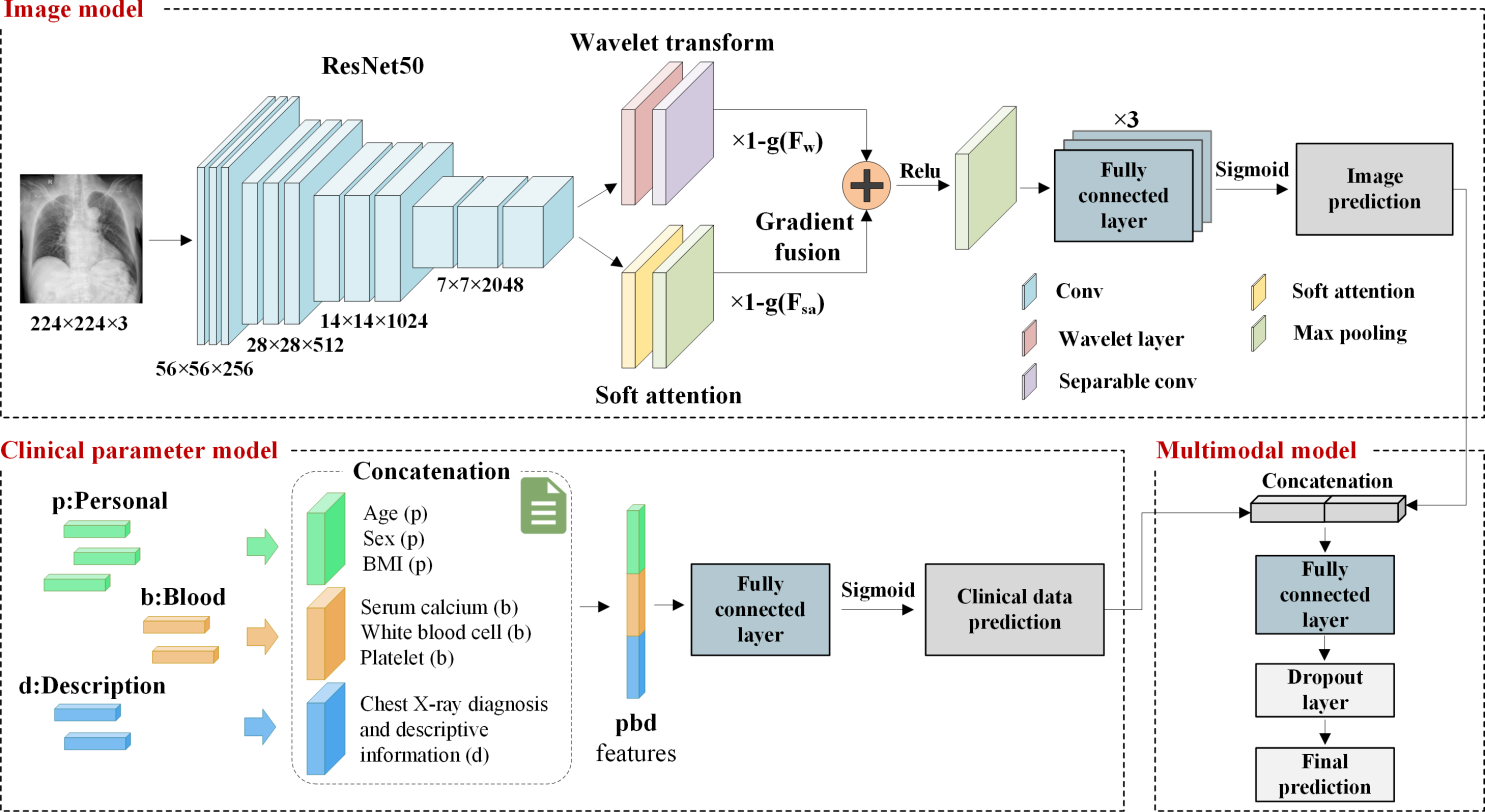
The proposed multimodal model framework. It includes 3 parts: image, clinical parameter, and multimodal models.

The framework of our proposed multimodal model is shown in Figure 1. After data preprocessing, we built an image model trained on X-ray images and a clinical parameter model trained on clinical data. The multimodal model then fused the prediction results of these two models using a probability fusion (PF) strategy [19]. The details of each model are described in the following sections.

#### Image Model

Transfer learning is a method that uses pretrained models to reduce the data and computational costs required for new tasks. In the field of medical imaging, methods that combine transfer learning with CNN models trained on large-scale datasets such as ImageNet have been widely adopted [28]. Our image model used a pretrained ResNet50 [29] as the backbone network for feature extraction of chest X-ray images. Subsequently, we applied the wavelet transform (WT) for multiscale analysis of the images to extract features at different scales that reveal subtle changes in bone structure in different images. The soft attention (SA) mechanism was used to capture specific appearance features and morphological regions in the images. Finally, we used a gradient-based feature fusion mechanism to effectively fuse these features to obtain the final image probabilistic output.

ResNet50 has excellent feature extraction capabilities, with a network depth of up to 50 layers, which could effectively capture rich hierarchical features in image data. Meanwhile, the residual learning structure adopted by ResNet50 effectively solved the problem of gradient vanishing during deep network training. In addition, it also had a lower number of parameters and computational costs, making it more practical for common hardware deployment scenarios in medical imaging analysis. For the binary classification task of osteoporosis, we fine-tuned our pretrained ResNet50 model. Specifically, considering that our classification task involved only 2 categories, which were significantly different from the 1000 categories in ImageNet shown in a previous study by Holste et al [30], we added a global maximum pooling layer and 3 fully connected layers with 1024, 512, and 256 neurons to the top of the pretrained model.

WT is a signal processing method based on multiscale analysis, which decomposes a signal into a weighted sum of multiple waves of different scales and frequencies through a mathematical transform. The WT has the advantage of multiscale and multiresolution and is capable of accurately extracting the local spatiotemporal distribution characteristics of the signal [31]. In our multimodal model, we used an efficient Haar WT method that extracted wavelet features from the output features of ResNet50. These features covered valuable information such as the sparsity of bone trabeculae and the thickness of the bone cortex. To reduce the parameters and computational effort of the model and to maintain the performance of the model, a depth-separable convolutional layer was introduced after the WT layer.

Inspired by the recent widespread application of the attention mechanism in deep neural networks [32], we introduced the attention mechanism into our model. Both SA [33] and hard attention are mechanisms that help models focus on key parts of input data. The hard attention mechanism involved discrete sampling at specific locations of input data, typically using reinforcement learning methods during training, as it involved nondifferentiable operations. In contrast, the SA mechanism was a probability distribution-based continuous attention method that assigned a weight to each position of the input data, and then weighted and summed the input based on these weights to obtain a context vector. The advantage of SA was that it was completely differentiable and could be trained end-to-end using standard gradient descent methods, making it more stable and efficient during the training process. In our study, we used a SA mechanism that assigned a weight to each input item, representing the degree of attention the model pays to that input item. This helped the model focus on key regions in X-ray images and generated descriptions that match the image content.

Specifically, we dynamically performed adaptive gradient fusion of wavelet features with SA features, using the normalized backpropagation gradients ${g(F}_{w})$ and $g(F_{sa})$ of $F_{w}$ and $F_{sa}$ as the basis for fusion weights, as shown in equation 1. A higher gradient means that the corresponding feature may have a negative impact on the model performance, and therefore it is given less weight in the fused features $F_{fuse}$, and vice versa. By introducing learnable weights, the feature fusion process was optimized to be more representative without increasing the parameters. This method ensured that important information was efficiently fused into the coded features before they were passed to the fully connected layer.


(1) $$F_{fuse}=\left(1-g\left(F_{w}\right)\right)\times F_{w}+\left(1-g\left(F_{sa}\right)\right)\times F_{sa}$$

#### Clinical Parameter Model

Our clinical parameter model includes important blood biochemical indicators such as white blood cell count, platelet count, serum calcium level, and fasting blood glucose and personal information including age, sex, and BMI, as well as chest X-ray diagnosis and descriptive information. Previously, most machine learning–based studies have relied mainly on clinical data, such as using prehospital information to predict mortality in patients with heart failure in intensive care units [34] or for predicting sepsis [35]. Algorithms such as random forest and XGBoost usually showed better performance than DL models for clinical data processing, and they did not require backpropagation during training; however, this made it difficult to directly combine these algorithms with DL models such as CNNs. Therefore, in our study, we chose to use a fully connected layer with 50 neurons to process these clinical data.

#### Multimodal Model

When training our multimodal model, we used the weights of the already trained baseline image model and clinical parameter model. We used a PF strategy in which both baseline models were run in inference mode, each independently producing a prediction. Our multimodal fusion mechanism then took the independent output probabilities of the image and clinical parameter models as inputs, concatenated them, and input them into a fully connected layer neural network with 32 neurons to generate the final prediction. During the training process, the learning rate started at each epoch and decreased in a multiplicative manner at the end of each epoch until a preset minimum was reached, and it took approximately 40 minutes to train the multimodal model once.

### Evaluation

We used 4 evaluation metrics to measure the performance of our model, including area under the curve (AUC) value, accuracy, sensitivity, and specificity. To obtain more accurate and less biased results, we adopted a 5-fold cross-validation method. In the hyperparameter settings, we used binary cross-entropy as the loss function and selected the Adam optimizer. We combined the ideas of grid search and random search, and tried multiple values such as $1e^{-4}$, $1e^{-5}$, and $1e^{-6}$ for the learning rate. For the dropout rate, we tried multiple values such as 0.2, 0.25, 0.3, 0.4, and 0.5. For batch size, we tried multiple values such as 16, 32, 64, and 128. The final determined learning rate was $1e^{-5}$, dropout was 0.25, and the batch size was 32. To effectively prevent overfitting of training data, we adopted an early stopping strategy based on validation loss and retained the optimal weights during the training process for recovery. Our model was implemented in Keras 2.4 and trained on a computer with an Intel i9-14900K 24-core CPU, 64 GB of RAM, and an NVIDIA RTX A6000 GPU card with 48 GB of memory.

## Results

### Patient Characteristics

**Figure 2.**
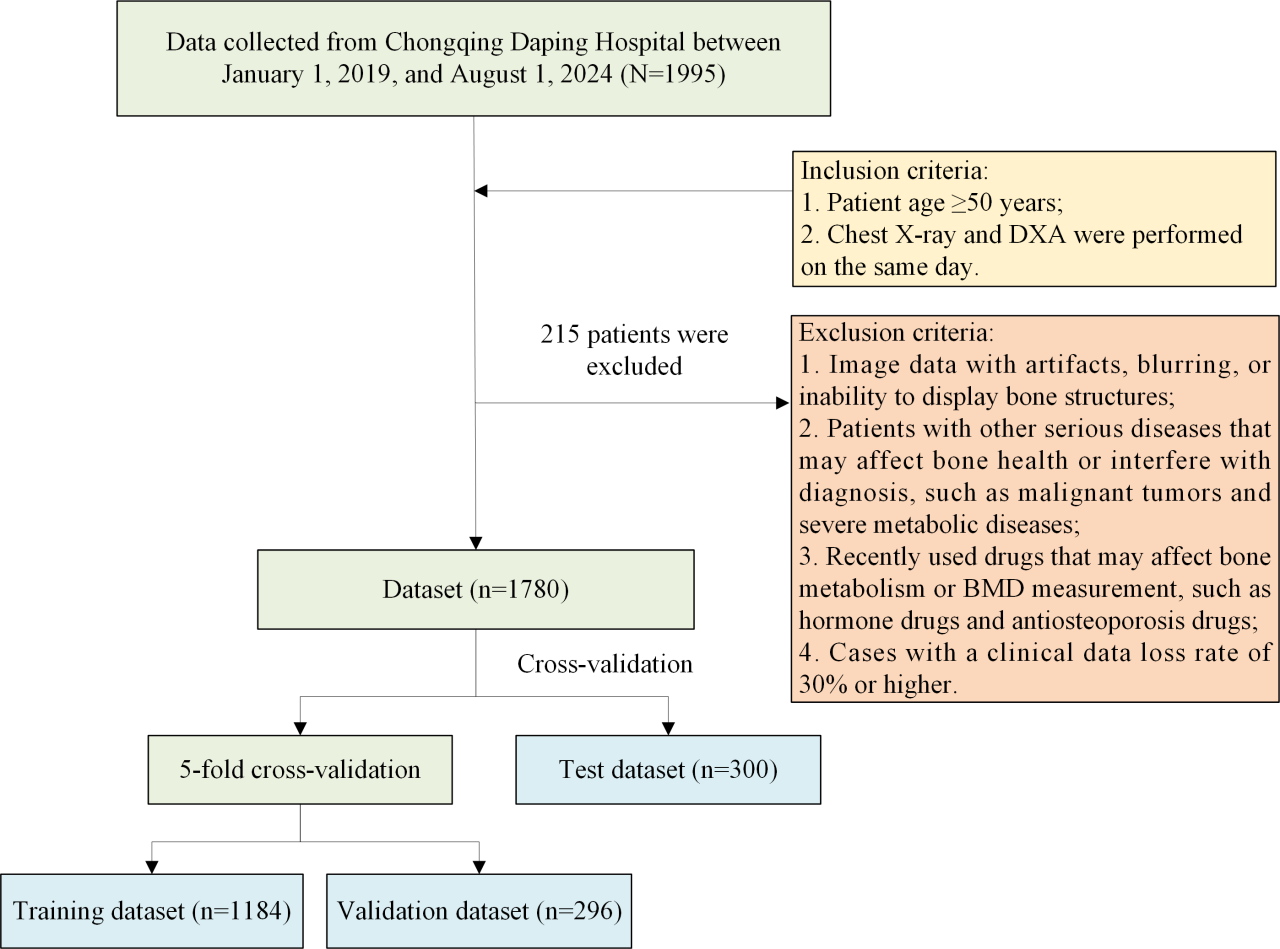
Patient inclusion process diagram. BMD: bone mineral density; DXA: dual-energy X-ray absorptiometry.

This study included a total of 1780 patients (as shown in the patient inclusion process flow diagram in Figure 2), with each patient corresponding to an anteroposterior position chest X-ray image. The average age of the patients was 69.46 (10.33) years, with an age range of 50 to 99 years. The statistical data of the training dataset, validation dataset, and test dataset are detailed in Table 1. These three datasets are almost identical in terms of sex and age distribution. All patients were divided into 2 groups: the osteoporosis group (990/1780, 55.62%) and the nonosteoporosis group (790/1780, 44.38%); 55.66% (659/1184), 56.4% (167/296), and 54.7% (164/300) of patients in the 3 datasets suffer from osteoporosis.

**Table 1.** Dataset description of 1780 cases of patients with osteoporosis.

Characteristics	Training dataset (n=1184)	Validation dataset (n=296)	Test dataset (n=300)
Age (y), mean (SD)	69.3 (10.34)	69.53 (10.72)	69.94 (10.28)
Sex, n (%)			
Male	495 (41.80)	119 (40.2)	120 (40.0)
Female	689 (58.20)	177 (59.8)	180 (60.0)
Presence of osteoporosis, n (%)			
Osteoporosis	659 (55.66)	167 (56.4)	164 (54.7)
Nonosteoporosis	525 (44.34)	129 (43.6)	136 (45.3)

### Overall Predictive Performance of the Multimodal Model

**Figure 3.**
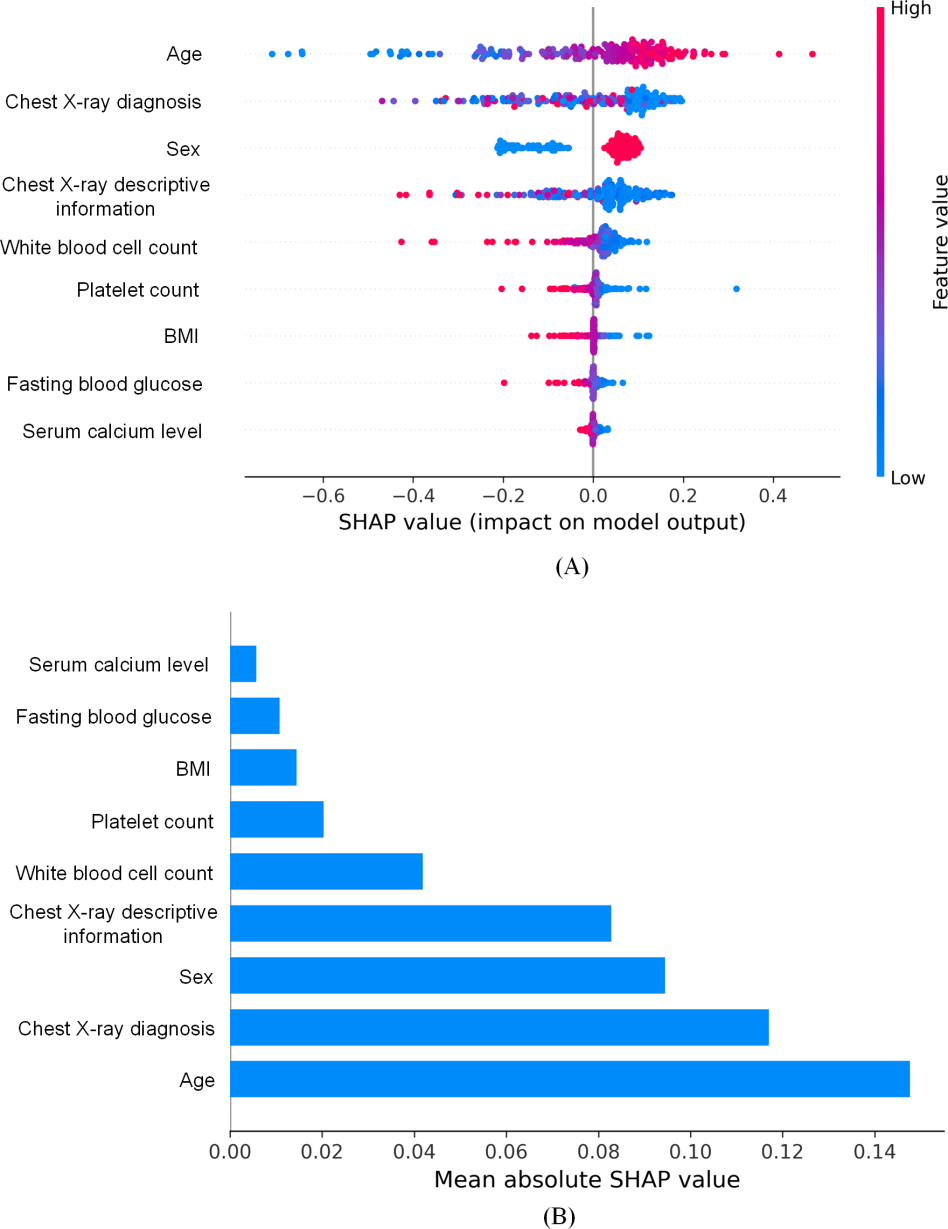
Shapley additive explanations (SHAP) visualization diagram. (A) Dot plot. (B) Bar plot.

In this study, we used the SHAP (Shapley additive explanations) method to analyze the feature importance of the model. The SHAP method helps us understand which features have a specific impact on the model’s decision-making process by calculating the contribution value (ie, SHAP value) of each feature to the model prediction. Figure 3 presents the results of the SHAP method, including a dot plot (Figure 3A) and a bar plot (Figure 3B). The dot plot displays the distribution of SHAP values for each feature, reflecting the direction and degree of the feature’s impact on the model’s prediction results. The bar plot summarizes the mean contribution of each feature to the model prediction. According to the analysis results, the features that contributed the most to the model prediction were age, chest X-ray diagnosis, sex, chest X-ray descriptive information, white blood cell count, platelet count, BMI, fasting blood glucose, and serum calcium level. These results indicated that the selected features were of great significance for model prediction, thereby improving the accuracy and reliability of the model.

**Figure 4.**
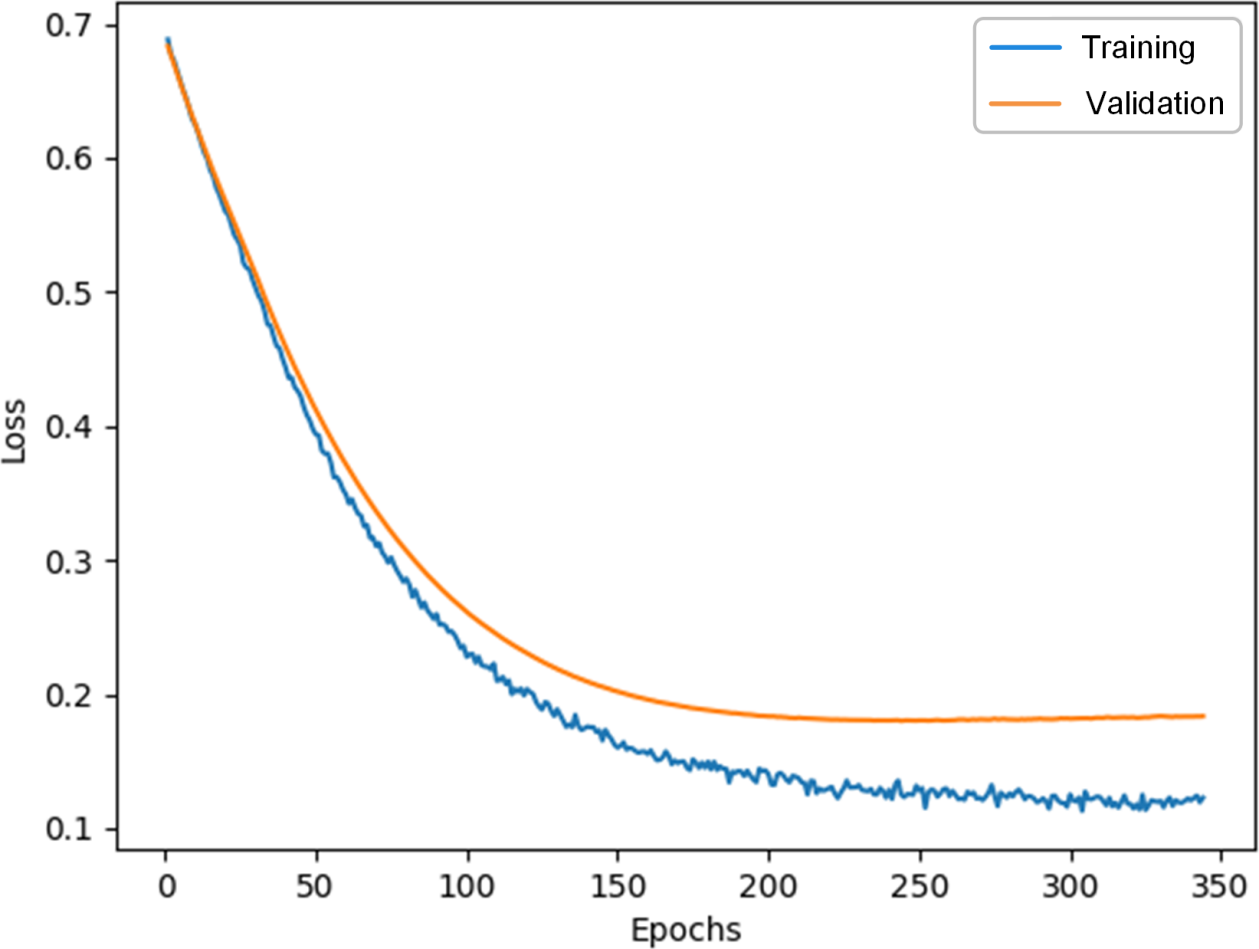
Training and validation loss curves of the multimodal model.

The training and validation loss curves of the multimodal model in this paper are shown in Figure 4, which intuitively demonstrate the convergence of the model. From Figure 4, it could be seen that as the training progresses, both the training loss and validation loss exhibited a decreasing trend and gradually stabilized, indicating that the model performs consistently on both the training and validation datasets and has good generalization ability.

To validate the superiority of the multimodal model proposed in this paper, Table 2 summarizes the performance of the model in the test dataset. First, to verify the performance of the ResNet50 pretrained backbone network used in the image model of this paper, we selected a conventional CNN model and two other pretrained models, Inception v3 and VGG16, for comparative analysis. Among them, the pretrained models ResNet50 and VGG16 used images of size 224×224 pixels as input, while the Inception v3 model used images of size 299×299 pixels. The image model corresponds to the pretrained ResNet50 model in Table 2. As shown in the table, all 4 image-based models showed good classification ability in the test dataset, with an average AUC value of 0.902. In comparison, our image model showed the optimal structural performance compared to the other 3 models. Specifically, our image model achieved the highest values in the 4 evaluation metrics of AUC value, accuracy, sensitivity, and specificity, which were 0.951, 89.32%, 89.82%, and 88.64%, respectively.

**Table 2.** The performance of models in the test dataset.

Model	AUC^a^	Accuracy, %	Sensitivity, %	Specificity, %
CNN^b^	0.832	77.91	83.54	69.75
Inception v3	0.909	80.74	77.66	84.96
VGG16	0.916	85.54	85.26	85.92
Image model	0.951	89.32	89.82	88.64
Clinical parameter model	0.884	80.81	78.29	84.46
Multimodal model	0.975	92.36	91.23	93.92

a AUC: area under the curve.

b CNN: convolutional neural network.

The multimodal model trained in this paper, which combines patient image and clinical parameter features, outperformed the image-only or clinical parameter–only models on all 4 evaluation metrics. Specifically, when compared with the model using only X-ray images, the multimodal model improved its AUC value from 0.951 to 0.975 (*P*=.004), accuracy from 89.32% to 92.36% (*P*=.045), sensitivity from 89.82% to 91.23% (*P*=.03), and specificity significantly from 88.64% to 93.92% (*P*=.008). Notably, the multimodal model effectively reduced the false positive rate and increased the specificity by 5.28% compared with the unimodal image model at the high-sensitivity operating point. We experimentally verified the superiority of the multimodal method in this paper compared to several other classification models.

**Figure 5.**
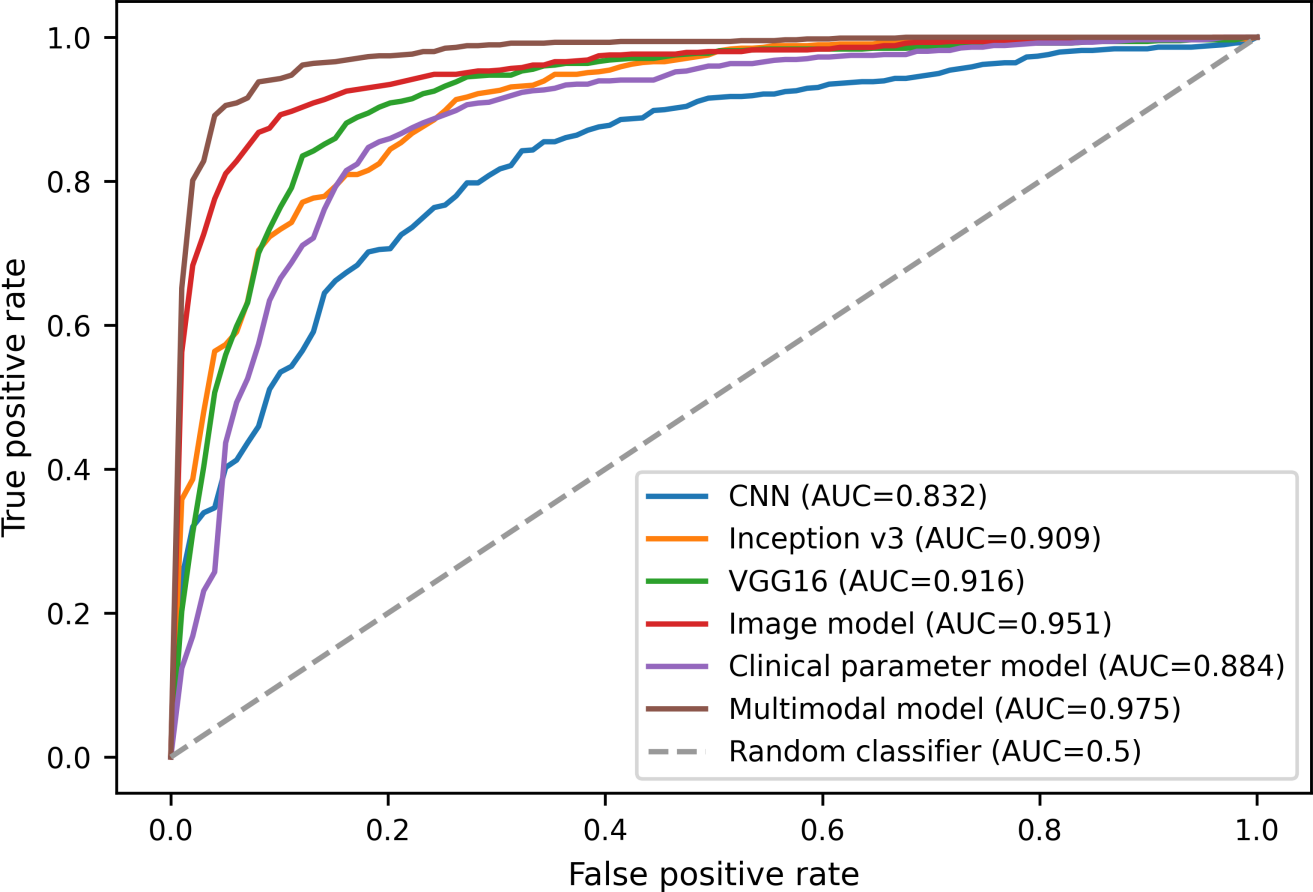
Receiver operating characteristic (ROC) variation curves in the test dataset. AUC: area under the curve; CNN: convolutional neural network.

**Figure 6.**
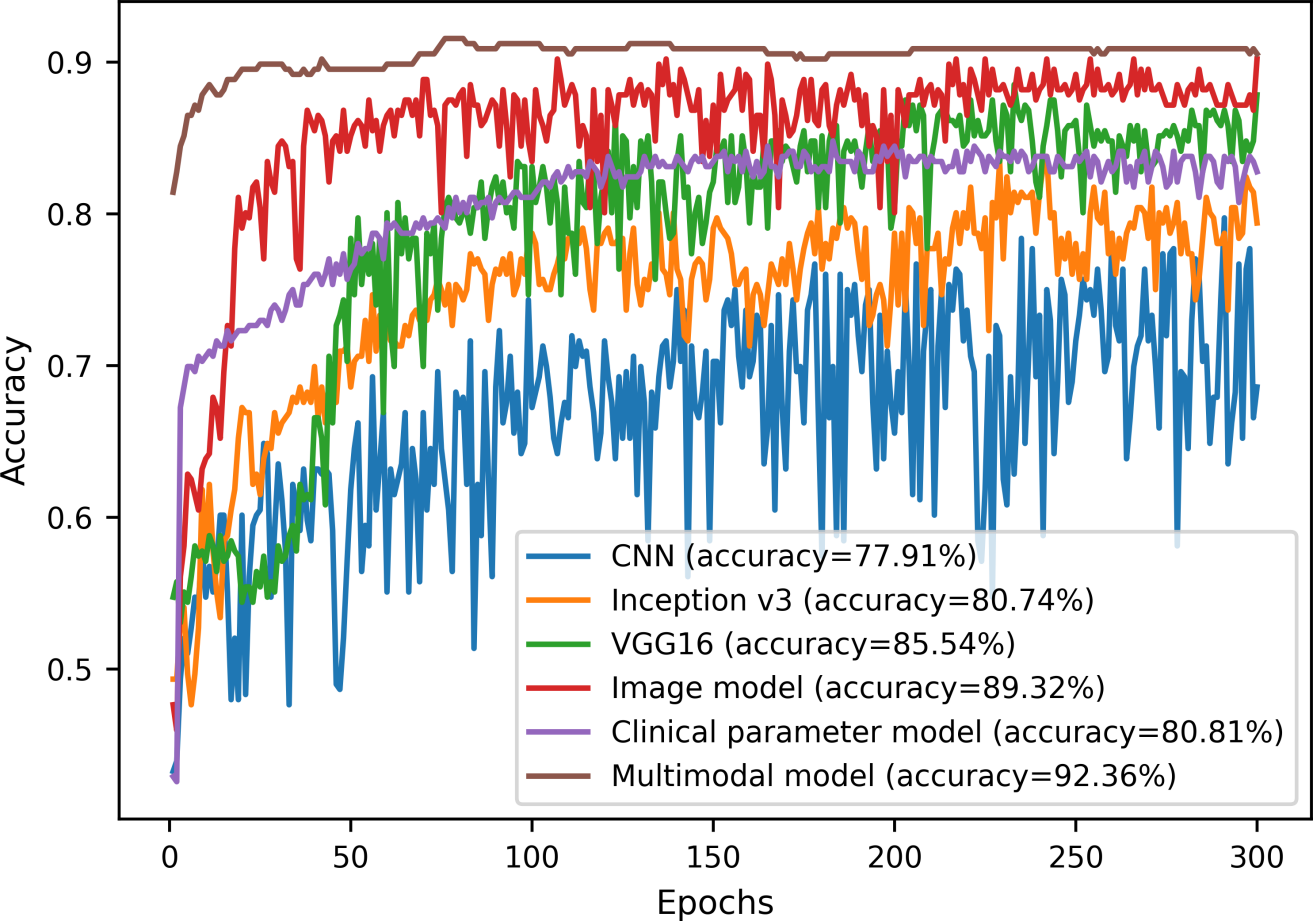
Accuracy variation curves on the test dataset. CNN: convolutional neural network.

The variation curves of receiver operating characteristic and accuracy for the image model, the clinical parameter model, and the multimodal model on the test dataset are shown in Figures5  6, respectively. In Figure 6, we show the results for only the first 300 epochs of each model. From the figure, the multimodal model exhibits the lowest false positive rate and the highest true positive rate, with an AUC value of 0.975, as well as an accuracy of 92.36%. In contrast, the CNN model has an accuracy of only 77.91% with an AUC value of 0.832, with a difference of more than 10% between the two models. In addition, the multimodal model proposed in this paper is the first to reach the convergence state and has the least fluctuation compared with other models.

**Figure 7.**
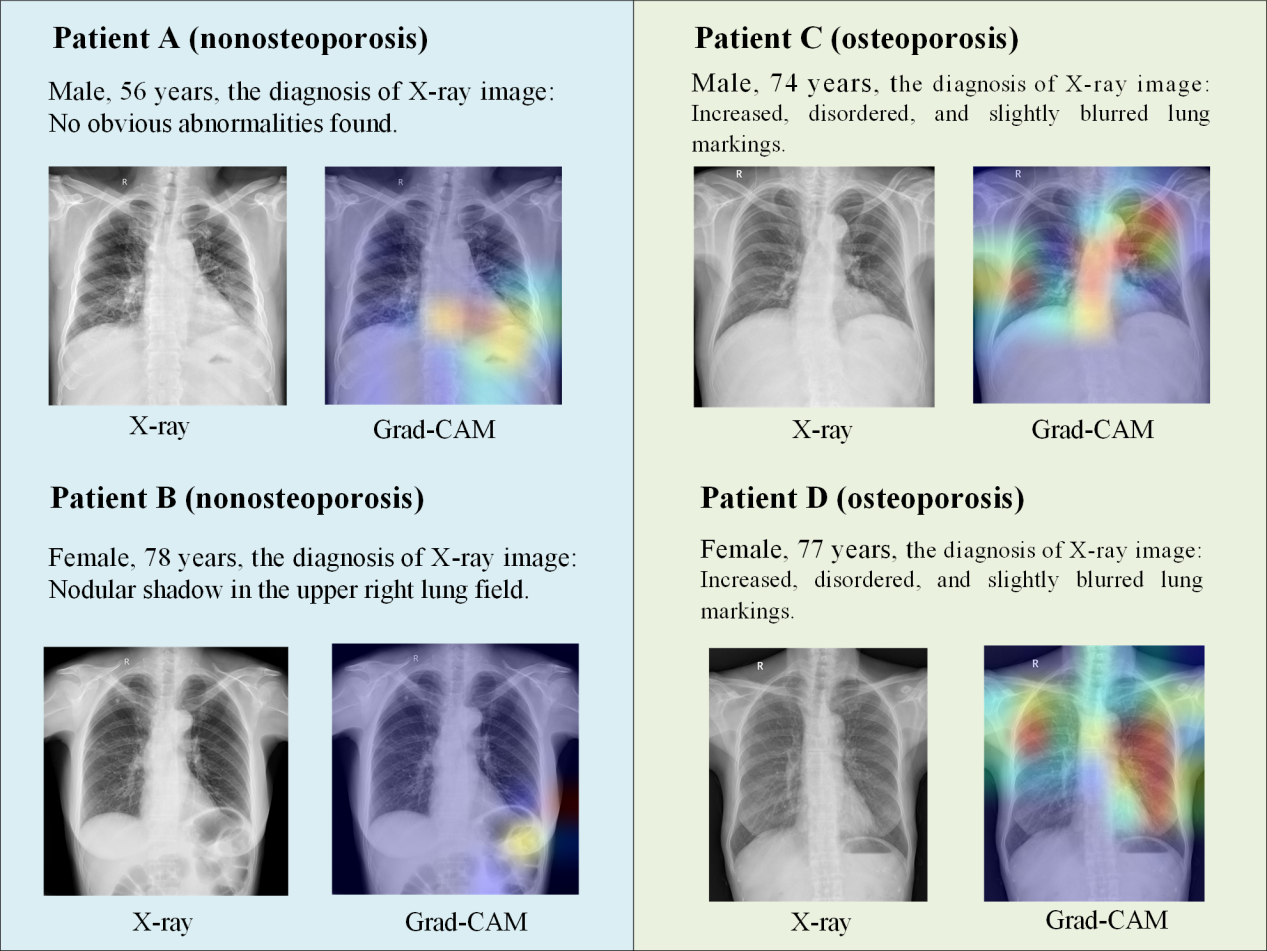
The visual interpretation of individual patients’ outcomes. We randomly selected data from 4 patients for interpretation. The images on the left side show the patients’ chest X-ray images, and the images on the right side show the corresponding gradient-weighted class activation mapping (Grad-CAM) heat maps. Areas that appear as bright red on chest X-ray images are considered to be the most important.

To visualize the attentional regions of the model, we used the gradient-weighted class activation mapping technique. Figure 7 shows the results of the visual interpretation of the multimodal model at the individual level. In patients with osteoporosis, as BMD decreases, a series of characteristic changes in bone structure occur, such as sparse trabeculae and thinning of cortical bone. The gradient-weighted class activation mapping heat map showed that the model mainly focused on areas such as the scapula, thoracic spine, ribs, and sternum. These areas are commonly vulnerable sites in osteoporosis, and their structural changes are closely related to the pathological characteristics of osteoporosis. For example, the thoracic and lumbar spine is a high-risk area for osteoporotic fractures, and a decrease in BMD can increase the risk of fractures. The model could more accurately predict osteoporosis by learning the imaging features of these key regions. The model’s attention to the scapula and ribs also had certain clinical significance. The changes in BMD of the scapula and ribs could indirectly reflect the overall bone condition of the chest, providing supplementary information for the diagnosis of osteoporosis. In addition, although anteroposterior chest X-rays could not fully cover areas such as the humeral head and lumbar spine, the model could still make effective predictions by focusing on key bone structures observable in the chest X-rays.

### Ablation Study

To verify the role of WT and SA mechanisms in our image model, we conducted an ablation study, which included (1) ResNet50, (2) ResNet50+WT, ResNet50+SA, and ResNet50+WT+SA.

In Table 3, it could be seen that the introduction of the WT and the SA mechanism based on the backbone network ResNet50 improved the performance of the model in all 4 evaluation metrics, especially in the specificity metric, where the improvement reaches 7.74%. The results of this ablation study demonstrated the ability of these two modules to enhance the model’s attention to key regions of the image and validate their effectiveness in our model.

**Table 3.** Optimal model configuration for the ablation study.

Model	AUC^a^	Accuracy, %	Sensitivity, %	Specificity, %
ResNet50	0.930	86.35	86.97	80.90
ResNet50+WT^b^	0.950	87.70	89.18	85.99
ResNet50+SA^c^	0.941	89.19	90.18	87.97
ResNet50+WT+SA	0.951	89.32	89.82	88.64

a AUC: area under the curve.

b WT: wavelet transform.

c SA: soft attention.

In addition, we experimented with different multimodal fusion strategies, and the results are shown in Table 4. As shown in the table, the strategy we used to merge the output probabilities before learning the final decision showed better performance compared to the two models that performed fusion by merging intermediate features.

**Table 4.** Performance comparison of different fusion strategies.

Fusion strategy	AUC^a^	Accuracy, %	Sensitivity, %	Specificity, %
FF^b^	0.969	91.49	90.99	92.16
LFF^c^	0.957	89.32	89.94	88.48
PF^d^	0.975	92.36	91.23	93.92

a AUC: area under the curve.

b FF: feature fusion.

c LFF: learning feature fusion.

d PF: probability fusion.

To verify whether the performance improvement of PF compared to feature fusion (FF) and learning FF (LFF) was statistically significant, we conducted a 2-tailed paired *t* test on accuracy, and the experimental data were obtained based on 5-fold cross-validation. The results showed that the *t* value of PF relative to FF was *t*_4_=4.2426, and the *P* value was .01; and the *t* value of PF relative to LFF was *t*_4_=2.9774, and the *P* value was .04. At a significance level of α=.05, the performance differences between PF and FF, as well as between PF and LFF, were statistically significant (*P*<.05). It could be seen that PF had the best performance among the 3 fusion methods in our experimental setup.

To improve the generalization performance and robustness of the model, we dynamically augmented the image data using a stochastic augmentation strategy. Comparing the model performance with and without the dynamic data augmentation technique, the results are shown in Table 5. In addition to the sensitivity metrics, the 3 metrics of AUC value, accuracy, and specificity were significantly improved after the introduction of dynamic data enhancement, especially the specificity, which was enhanced by 15.2%, greatly improving the accuracy of the model in identifying nonosteoporosis and reducing the rate of false alarms.

**Table 5.** Performance comparison of the image model with and without dynamic data augmentation.

Data augmentation	AUC^a^	Accuracy, %	Sensitivity, %	Specificity, %
Without	0.934	84.32	92.28	73.44
With	0.951	89.32	89.82	88.64

a AUC: area under the curve.

## Discussion

### Principal Findings

Although DL has made substantial progress in the field of medical imaging, it still focuses primarily on the imaging data themselves, largely ignoring the richness of information available in the clinic. Our experimental results show that the multimodal model fusing chest X-ray images and clinical data can improve the accuracy of deep neural networks in predicting osteoporosis. Through the ablation study, we validated the WT and the SA mechanism to enhance the performance of the model by capturing specific appearance features in the images, and also confirmed the advantages of dynamic data augmentation and PF strategies in a multimodal model.

Osteoporosis, as a major health disease, is becoming increasingly prominent in the aging population. Predictive studies of osteoporosis not only help us to gain a deeper understanding of the disease’s trends and risk factors, but also provide a scientific basis for the development of effective prevention and treatment strategies. The classification model we designed is a binary classification model based on chest X-ray images and clinical data, aiming to classify outcomes into 2 categories: osteoporosis and nonosteoporosis. The results show that multimodal data can be used to distinguish patients with osteoporosis from those with normal BMD, and that the method we used requires a small amount of training data and a short processing time. As a disease screening method, the model in this paper has high sensitivity and helps to reduce the false negative rate. The model can be used both prospectively by radiologists and retrospectively by radiologists or even nonradiologists, which is important for the timely identification and treatment of patients with osteoporosis.

We downsample the X-ray image to 224×224 pixels to meet the input requirements of the ResNet50 model, while the Inception v3 architecture used for comparison requires downsampling the image to 299×299 pixels. Interestingly, although Inception v3 has a higher input resolution, its performance is not significantly better than ResNet50. This discovery suggests that simply increasing input resolution may not significantly improve model performance. This may be related to factors such as the complexity of the model architecture and the characteristics of the target task.

Since most relevant studies [9,11,14,36] currently use private datasets, it is difficult to find data that is identical or highly comparable to our dataset for direct comparison. Therefore, we mainly started from the aspects of dataset size, model architecture, and key results to compare the existing paper on osteoporosis screening based on X-ray using DL methods, as shown in Table 6. Although the research by Mao et al [14] bears some resemblance to ours, substantial differences remain. They only analyzed X-rays and clinical data using a single CNN model. And based on this, we designed a multimodal model to process X-rays and clinical data separately. This innovative model architecture not only enhances the model’s ability to process different types of data but also fully explores and uses the inherent correlation and value of multisource data, thereby achieving more accurate screening for osteoporosis.

**Table 6.** Comparison with related works.

Reference	Dataset	Model	Key results
Lee et al [9]	334 spine X-rays	DL^a^ and machine learning	AUC^b^: 0.74 Accuracy: 0.71 Sensitivity: 0.81 Specificity: 0.60
Jang et al [11]	13,026 chest X-rays	DL	AUC: 0.91 Accuracy: 82.40%
Mao et al [14]	5652 lumbar spine X-rays and clinical data	CNN^c^	AUC: 0.909‐0.937
Wani et al [36]	381 knee X-rays	DL and transfer learning (AlexNet)	Accuracy: 91.1%
This study	3645 chest X-rays and clinical data	Transfer learning (ResNet50) and multimodal model	AUC: 0.975 Accuracy: 92.36% Sensitivity: 91.23% Specificity: 93.92%

a DL: deep learning.

b AUC: area under the curve.

c CNN: convolutional neural network.

In this study, our proposed multimodal model demonstrated high accuracy in predicting osteoporosis, indicating its potential clinical application value. However, in the process of transforming the model from a laboratory environment to practical clinical applications, the feasibility of deployment also needs to be considered. To ensure that the model can run effectively in practical clinical scenarios, we recommend integrating it with the hospital’s picture archiving and communication system and electronic medical record systems. This can automatically acquire and process imaging and clinical data, thereby reducing the burden on technicians in preprocessing work. In addition, the predicted results of the model can be automatically integrated into the patient’s electronic medical record for doctors to review and reference at any time. This model can run on regular servers or cloud platforms with low hardware requirements, making it suitable for environments with limited resources.

### Limitations

Our study has some limitations. First, we have not systematically analyzed the performance of the model under different races, age groups, or imaging protocols, which may introduce bias and affect the fairness and universality of the model. Given this, we plan to include more diverse population data and data obtained from different imaging protocols in future studies to evaluate and improve the performance of the model in various populations. Second, the coverage range of anteroposterior chest X-rays has certain limitations and cannot fully include key areas such as the humeral head and lumbar spine. The insufficient visual coverage may affect the diagnostic accuracy of the model for certain patient populations, especially those with substantially pathological features in uncovered areas. The plan is to introduce lateral chest X-rays to address this problem. Third, the data for this study came from a single institution, Chongqing Daping Hospital, which may limit the generalizability of the model in different populations. External validation is crucial to confirm the robustness of the model in different populations. Future research plans will validate the model on multiple datasets, which will help evaluate its generalization ability. Finally, although this paper used the WT and SA mechanism to enhance the model and conduct the ablation study, these components were not compared with traditional feature extraction techniques such as Gabor filters, local binary patterns, or radiomics-based features. We plan to supplement the fusion experiments of Gabor filters, local binary patterns, and other methods with deep features in future experimental work. In addition, to further explore and validate the existence of better fusion strategies, we plan to incorporate transformer-based cross-modal attention models in future research and compare their performance with PF strategies. The transformer architecture demonstrates powerful capabilities in handling multimodal data, particularly in capturing complex relationships between different modalities.

### Conclusions

In osteoporosis prediction, our proposed multimodal model fusing chest X-ray images and clinical data improves the prediction accuracy, with AUC value, accuracy, sensitivity, and specificity metrics exceeding 90%, which all indicate significantly better performance than models relying only on images or clinical data. The model in this paper is expected to be an effective tool that clinicians can use to screen for opportunistic osteoporosis without increasing radiation exposure or additional costs. It is particularly suitable for patients who have had a chest X-ray but have not undergone DXA. With this model, clinicians can detect osteoporosis symptoms early and provide timely treatment interventions to prevent further bone loss.

## Abbreviations

AUC: area under the curve
BMD: bone mineral density
CNN: convolutional neural network
DL: deep learning
DXA: dual-energy X-ray absorptiometry
FF: feature fusion
LFF: learning feature fusion
PF: probabilistic fusion
SA: soft attention
SHAP: Shapley additive explanations
WT: wavelet transform
